# Density Functional Theory Provides Insights into β-SnSe Monolayers as a Highly Sensitive and Recoverable Ozone Sensing Material

**DOI:** 10.3390/mi15080960

**Published:** 2024-07-27

**Authors:** Jiayin Wu, Zongbao Li, Tongle Liang, Qiuyan Mo, Jingting Wei, Bin Li, Xiaobo Xing

**Affiliations:** 1 Department of Engineering Technology, Guangdong Open University, Guangzhou 510091, China; wujiayin@m.scnu.edu.cn (J.W.); jtwei@gdrtvu.edu.cn (J.W.); bli@gdrtvu.edu.cn (B.L.); 2 Centre for Optical and Electromagnetic Research, South China Academy of Advanced Optoelectronics, South China Normal University, Guangzhou 510006, China; 3 Ministry of Education Key Laboratory of Textile Fiber Products, School of Materials Science and Engineering, Wuhan Textile University, Wuhan 430220, China; 4 School of Materials and Chemistry Engineering, Tongren University, Tongren 554300, China; 5 School of Artificial Intelligence, Guangdong Vocational College of Post and Telecom, Guangzhou 510630, China; liangtongle@gupt.edu.cn; 6 Big Data Engineering College, Kaili University, Kaili 556011, China; 2014020983@kluniv.edu.cn

**Keywords:** β-SnSe monolayer, ozone, sensor, DFT, sensitivity

## Abstract

This study explores the potential of β-SnSe monolayers as a promising material for ozone (O_3_) sensing using density functional theory (DFT) combined with the non-equilibrium Green’s function (NEGF) method. The adsorption characteristics of O_3_ molecules on the β-SnSe monolayer surface were thoroughly investigated, including adsorption energy, band structure, density of states (DOSs), differential charge density, and Bader charge analysis. Post-adsorption, hybridization energy levels were introduced into the system, leading to a reduced band gap and increased electrical conductivity. A robust charge exchange between O_3_ and the β-SnSe monolayer was observed, indicative of chemisorption. Recovery time calculations also revealed that the β-SnSe monolayer could be reused after O_3_ adsorption. The sensitivity of the β-SnSe monolayer to O_3_ was quantitatively evaluated through current-voltage characteristic simulations, revealing an extraordinary sensitivity of 1817.57% at a bias voltage of 1.2 V. This sensitivity surpasses that of other two-dimensional materials such as graphene oxide. This comprehensive investigation demonstrates the exceptional potential of β-SnSe monolayers as a highly sensitive, recoverable, and environmentally friendly O_3_ sensing material.

## 1. Introduction

Ozone (O_3_), as a trace gas in the atmosphere, possesses a strong oxidizing capacity that can irritate the respiratory tract and eyes, especially affecting children, the elderly, and those with respiratory diseases. When present above certain levels, ozone can adversely impact physical and mental health. Monitoring ozone levels is crucial for maintaining its relative stability in the atmosphere and ensuring air quality.

Currently, ozone monitoring primarily relies on two methods: spectrophotometry and chemiluminescence. The former utilizes ultraviolet characteristics to measure ozone concentration based on absorption and wavelength in a medium, while the latter determines concentration based on the intensity of emitted light. However, both methods require laboratory settings, are time-consuming, and incur high costs, making continuous real-time monitoring of ozone levels challenging.

Nanostructured materials have emerged as promising candidates for ozone sensing due to their inherent advantages. Their high surface-to-volume ratio facilitates enhanced gas–solid interactions, promoting efficient ozone adsorption and subsequent sensing mechanisms. One-dimensional nanotubes, exemplified by pristine single-walled aluminum nitride nanotubes [1], and zero-dimensional nanocages, including Ni-doped Zn_12_O_12_ [2], B_12_N_12_ [3], BN fullerene-like structures [4], AlB_11_N_12_, GaB_11_N_12_, and Al_12_N_12_ [5], have shown particular promise for ozone detection. These nanostructures possess hollow interiors and abundant unsaturated surface atoms, both of which contribute to their ozone sensing capabilities.

Leveraging the excellent adsorptive properties of TiO_2_, nanomaterials based on TiO_2_, such as quantum dots of TiO_2_ [6], N-doped TiO_2_-supported Au nanocomposites [7], TiO_2_/WSe_2_ heterostructures [8], and nitrogen-doped TiO_2_/ZnO, have been employed to augment ozone adsorption. Additionally, the ozone sensing performance of graphene-based two-dimensional materials, including B-doped graphene [9], Pt-decorated graphene [10], and graphene oxide [11], has been studied.

In addition to these burgeoning two-dimensional materials, tin-based materials have garnered significant attention for their potential in ozone sensing applications. Boron doping has been shown to enhance charge transfer from stanene to ozone molecules, positioning it as a promising candidate for the development of sensitive ozone sensors [12]. Furthermore, the formation of heterojunctions, particularly by integrating stanene with materials like TiO_2_, has been investigated to exploit synergistic effects that further amplify ozone sensing performance [13]. These advancements underscore the innovative strides being made in the realm of material science for environmental monitoring. Furthermore, SnS has been identified as exhibiting a high degree of efficacy in interacting with ozone, positioning it as a promising candidate for chemical sensing in ozone detection methodologies [14].

β-SnSe (hereafter referred to as SnSe), a layered transition metal chalcogenide with a graphene-analogous structure, has attracted considerable interest owing to its extraordinary attributes and diverse potential applications. At elevated temperatures, SnSe exhibits an exceptionally low lattice thermal conductivity, rendering it a promising material for thermoelectric energy conversion [15,16,17]. Notably, SnSe possesses desirable characteristics, including chemical stability, non-toxicity, and abundant terrestrial reserves, making it suitable for large-scale applications and sustainable development. SnSe possesses a tunable band gap and superior light absorption properties, particularly in its monolayer form, where it demonstrates significant photoresponsiveness and exceptional photoelectric performance [18]. In terms of gas sensing, SnSe has demonstrated strong chemical adsorption towards various gas molecules, such as methanol (CH_3_OH), oxygen (O_2_), nitrogen dioxide (NO_2_), and sulfur dioxide (SO_2_), making it a compelling candidate for gas detection applications [19].

In this study, the potential performance of monolayer SnSe as an O_3_ sensing material was systematically investigated using density functional theory (DFT) combined with the non-equilibrium Green’s function (NEGF) method. Theoretical calculations provided an in-depth analysis of the adsorption characteristics of O_3_ molecules on the surface of monolayer SnSe, involving adsorption energy, band structure, density of states (DOSs), differential charge density, and Bader charge analysis. These calculations revealed the interaction mechanism and electron transfer process between the monolayer SnSe and O_3_ molecules, elucidating its fundamental sensing properties as an O_3_ gas detector. Additionally, this work specifically considered the recoverability of monolayer SnSe in practical applications, evaluating the recovery time after O_3_ adsorption through computational assessments. Furthermore, the response sensitivity of monolayer SnSe to O_3_ gas was quantitatively explored based on current-voltage (I–V) characteristic simulations. This research demonstrates the immense potential of monolayer SnSe as a highly sensitive, recoverable, and environmentally friendly O_3_ sensing material.

## 2. Materials and Methods

First-principle calculations were performed using the Vienna Ab initio Simulation Package (VASP) 5.4 within the framework of DFT [20,21]. The exchange-correlation energy was treated with the generalized gradient approximation (GGA) in the form of the Perdew–Burke–Ernzerhof (PBE) functional [22]. To accurately account for the potential weak interactions, such as van der Waals forces, between the molecules and the substrate, the DFT-D3 correction scheme was further employed [23]. Spin polarization effects were also considered in the calculations.

Specifically, a 4 × 4 supercell system comprising 64 atoms was constructed to simulate the two-dimensional structure of an SnSe monolayer. An energy cutoff of 400 eV was set to ensure calculation accuracy. During the structural optimization, the maximum force on each atom was required to be lower than 0.02 eV/Å, and the total energy convergence criterion was also set to 10^−5^ eV to ensure sufficient accuracy in the structural optimization. Considering the effects of periodic boundary conditions, a 20 Å vacuum layer was established in the perpendicular direction to prevent interactions between adjacent periods. For Brillouin zone sampling, a Monkhorst–Pack grid of 4 × 4 × 1 was utilized. 

The adsorption energy (E_ad_) of the O_3_ molecule on the SnSe monolayer surface was calculated using the following equation:

(1) $$\begin{array}{c} E_{ad}=E_{total}-E_{SnSe}-E_{O_{3}} \end{array}$$

where E_total_ is the total adsorption energy of the optimized systems after O_3_ adsorption on the SnSe monolayer, E_SnSe_ is the energy of the optimized pristine SnSe monolayer, and E_O_3__ denotes the energy of the O_3_ molecule involved. 

The current-voltage characteristics (I–V curves) were conducted using the tranSiesta module within the Siesta 4.1.5 software package, in conjunction with NEGF theory [24]. Utilizing the Landauer–Büttiker formula [25], the electrical currents across the material under varying voltages were determined:

(2) $$\begin{array}{c} I\left(V_{b}\right)=\frac{2e}{h}\int_{\mu_{R}}^{\mu_{L}}T\left(E,V_{b}\right)\left[f\left(E-\mu_{L}\right)-f\left(E-\mu_{R}\right)\right] \end{array}$$

Here, I signify the electric current traversing the contact electrode at the bias voltage (V_b_). The terms μ_L_ and μ_R_ represent the electrochemical potentials of the left and right electrodes, respectively. $T\left(E,V_{b}\right)$ denotes the transmission coefficient at voltage V_b_ and energy E. The $f\left(E-\mu_{L}\right)$ accounts for the Fermi–Dirac distribution function of the left and right electrodes at energy E. 

Additionally, the VESTA 3.90.0a software was used to analyze the differential charge density distribution of the system before and after adsorption, as well as the optimized geometric configuration [26]. Finally, the vaspkit 1.3.3 tool was employed for effective post-processing and analysis of the raw computational data [27].

## 3. Results and Discussion

### 3.1. Structures

**Figure 1:**
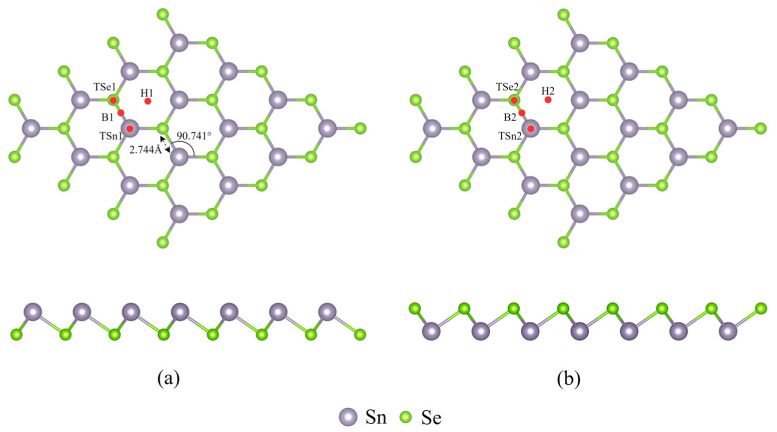
The structure and adsorption position of the SnSe monolayer: (**a**) Sn side; (**b**) Se side.

As depicted in Figure 1, the SnSe monolayer comprises two facets: the tin (Sn) side and the selenium (Se) side. On the Sn side, the O_3_ molecule may occupy four potential sites: atop the Sn atom (TSn1), atop the Se atom (Tse1), on the hollow site (H1), or on the bridge site (B1) between Sn and Se bonds. Similarly, on the Se side, the O_3_ molecule may be located atop the Sn atom (Tsn2), atop the Se atom (Tse2), on the hollow site (H2), or on the bridge site (B2) between Sn and Se bonds. Upon adsorption to the surface, the O_3_ molecule may approach in various orientations: with the lateral oxygen atoms near the surface and the central oxygen atom farther away, with a perpendicular arrangement to the SnSe surface; with the central oxygen atom near the surface and the lateral oxygen atoms farther away, with a perpendicular arrangement to the SnSe surface; or with the plane formed by the three oxygen atoms parallel to the SnSe surface. Energy calculations for different configurations indicate that the most stable and energetically favorable configuration is where the O_3_ molecule bridges between the Sn and Se bonds, with the lateral oxygen atoms approaching the surface and the central oxygen atom remaining farther away, forming a perpendicular orientation to the Sn side of the SnSe surface.

**Figure 2:**
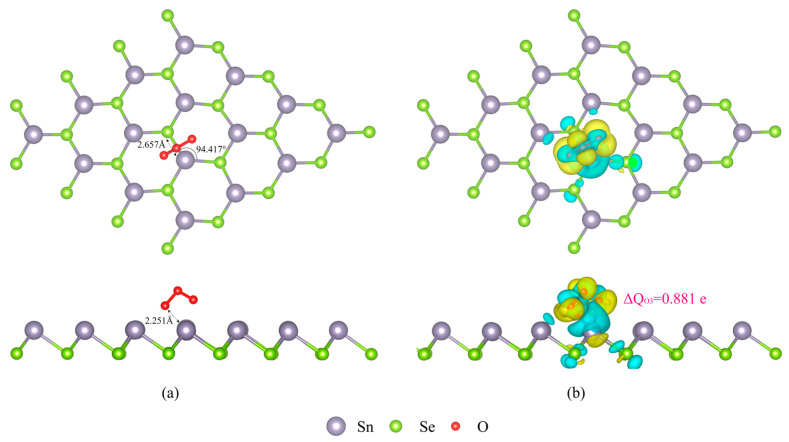
The most stable configuration (**a**) and differential charge density (**b**) of O_3_-adsopred SnSe monolayer.

As plotted in Figure 2a, the O_3_ molecule induces significant reconstruction of the SnSe monolayer surface during the adsorption process. Before adsorption, the Sn-Se bond length is approximately 2.744 Å, with a bond angle of 90.741°. However, upon O_3_ adsorption, the Sn atom beneath the adsorption site experiences a strong attractive force from the oxygen atoms, causing it to move closer to the oxygen atoms. This results in a contraction of the Sn-Se bond length to 2.657 Å, while the bond length between the Sn atom and the adjacent Se atoms elongates to 2.769 Å. Consequently, the Sn-Se-Se bond angle expands to 97.417°, reflecting the substantial impact of O_3_ adsorption on the SnSe surface structure. In the adsorption configuration, the distance between the closest oxygen atom of the O_3_ molecule and the Sn surface is 2.251 Å, suggesting the possibility of a strong adsorption interaction between Sn and the O_3_ molecule.

The calculated adsorption energy of the O_3_ molecule on the SnSe monolayer is −1.826 eV. This negative value clearly indicates that the O_3_ molecule adsorption on the SnSe surface is a thermodynamically spontaneous process that can proceed without the input of additional external energy. In other words, SnSe exhibits a strong affinity and easy adsorption for O_3_ molecules and can effectively capture O_3_ molecules at room temperature. Therefore, it is a promising candidate material for room-temperature O_3_ gas sensors.

### 3.2. Electronic Properties

In the in-depth exploration of the intrinsic mechanism behind the O_3_ gas sensing performance of monolayer SnSe, computational analysis was conducted on the electronic structure and band characteristics of monolayer SnSe before and after O_3_ adsorption. The Fermi level is adjusted to the zero point, and the band structure near the Fermi level, which is crucial for semiconductor properties, is specifically presented within the energy range of −4 eV to 4 eV.

**Figure 3:**
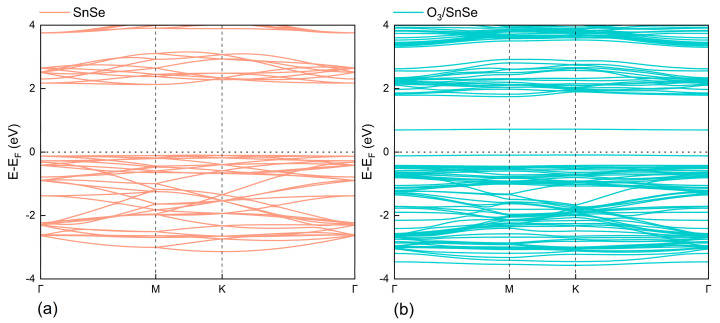
Band structures of pristine (**a**) and O_3_-adsorbed (**b**) SnSe monolayers.

The pristine SnSe monolayer is calculated to exhibit a theoretical band gap of 2.228 eV, as illustrated in Figure 3a, which is in close agreement with the previously reported theoretical value of 2.22 eV [16]. Upon O_3_ molecule adsorption on the SnSe monolayer surface, its electronic structure undergoes significant reconstruction. The adsorption process introduces new impurity energy levels near the Fermi level, which originate from the valence band maximum (VBM) and conduction band minimum (CBM) of the SnSe monolayer. These impurity energy levels directly influence the original band structure of SnSe, as seen in Figure 3b, leading to a remarkable decrease in the band gap to 0.790 eV.

This significant band gap narrowing implies that the conductivity of the SnSe monolayer is greatly enhanced after O_3_ adsorption. The newly formed impurity energy levels allow electrons in the valence band to jump to the conduction band at a lower energy threshold. Compared to the state before O_3_ adsorption, the activation energy required for electron migration is significantly reduced. Such changes in the electronic structure greatly improve the sensitive response efficiency of the SnSe monolayer to external stimuli as an O_3_ gas sensor.

**Figure 4:**
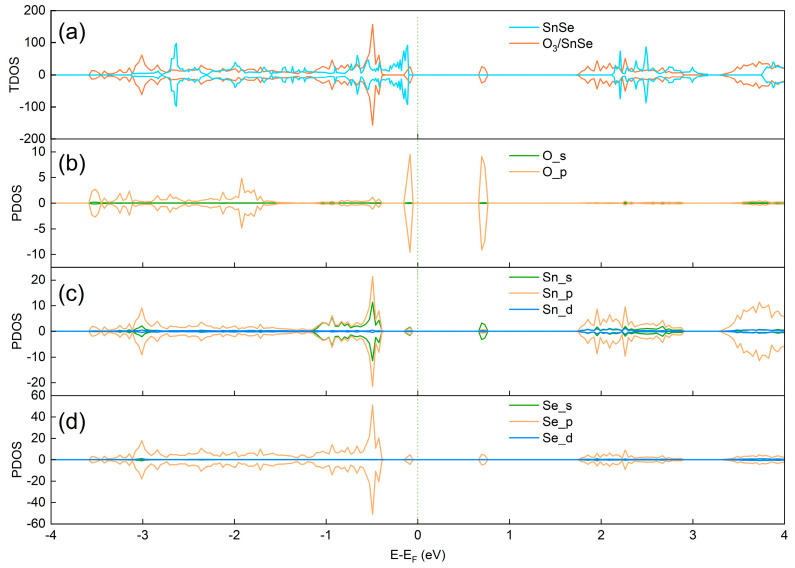
Total and partial DOSs of SnSe monolayer: (**a**) TDOSs and PDOSs of (**b**) O, (**c**) Sn, (**d**) Se (Fermi level is set as 0 eV).

Figure 4 delineates the total density of states (TDOSs) and partial density of states (PDOSs), where the adsorption of O_3_ on SnSe introduces hybridization energy levels flanking the Fermi level. The TDOSs analysis reveals a pronounced symmetry in the DOS curves for both spin-up and spin-down configurations, indicating the non-magnetic nature of the adsorption structure. Notably, significant peaks at energy values of −0.085 eV and 0.697 eV are observed, predominantly originating from the p orbitals of oxygen atoms.

At −0.085 eV, the oxygen-dominated absorption peak triggers peak responses in the s and p orbitals of Sn atoms and the p orbitals of Se atoms at the same energy level, unveiling a robust orbital hybridization indicative of changes in electron cloud distribution and energy level rearrangement during adsorption. Similarly, at 0.697 eV, oxygen atoms induce corresponding peaks in the s orbitals of Sn atoms and the p orbitals of Se atoms. These enhancements in electron density at specific energy levels directly affect the material’s band structure, particularly introducing impurity levels near the top of the valence band and altering the electronic transport characteristics near the bottom of the conduction band, thereby modifying the material’s electrical conductivity and reactive properties post-adsorption.

To elucidate the bonding characteristics of adsorbed atoms on the SnSe monolayer surface, the charge density differential was computed using the following equation:

(3) $$∆\rho =\rho_{SnSe+O_{3}}-\rho_{SnSe}-\rho_{O_{3}}$$

where Δρ represents the charge density differential, ρ_SnSe+O_3__ is the charge density of the adsorption system, ρ_SnSe_ is the charge density of the SnSe monolayer surface, and ρ_O_3__ is the charge density of the adsorbed O_3_ molecule.

Figure 2b presents three-dimensional isosurface plots of the differential charge density, where yellow regions represent electron accumulation and blue regions represent electron depletion. As seen in Figure 2b, when the O_3_ molecule is adsorbed on the SnSe monolayer surface, a significant electron depletion region appears on the Sn atom side opposite to the O atom, while a large amount of electron accumulation occurs around the O atom. This indicates that the O atom gains electrons from the Sn atom. Additionally, Figure 2b also reveals that the surface of the three Se atoms bonded to the O_3_ molecule also exhibits electron depletion regions, further confirming the transfer of electrons from the SnSe monolayer to the O_3_ molecule during the adsorption process.

Bader charge analysis reveals that the O_3_ molecule gains a net charge of 0.881 electron units, while the bonded Sn atom loses 1.200 electron units. This result provides strong evidence for a significant charge exchange phenomenon occurring between the O_3_ molecule and the SnSe surface, suggesting the high adsorption stability of the O_3_ molecule on the SnSe surface.

### 3.3. Recovery Time

Recovery time is a critical metric for evaluating the reversibility of sensors, referring to the time required to desorb target gas molecules from the surface of the sensing material. Recovery time τ is typically inversely related to adsorption energy and can be estimated through transition state theory:

(4) $$\begin{array}{c} \tau =exp\left(\frac{-E_{ad}}{k_{B}T}\right)/\omega \end{array}$$

where E_ad_ is the adsorption energy, k_B_ is the Boltzmann constant, and T is the temperature. ω is the attempt frequency, assumed to be 10^13^ s^−1^ [28].

**Figure 5:**
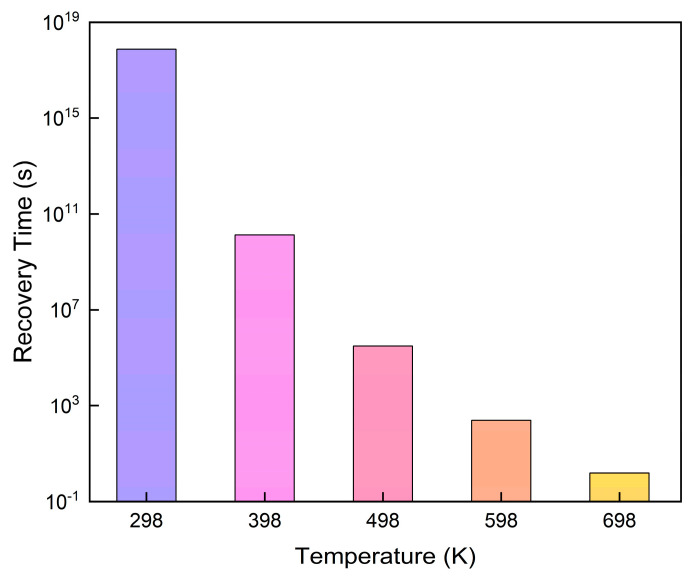
The recovery time (τ) for O_3_ adsorbed on SnSe monolayers at different temperatures (T).

Figure 5 illustrates how temperature affects the recovery time of the SnSe monolayer following O_3_ molecule adsorption. The recovery time for the SnSe monolayer following O_3_ adsorption can reach 7.61 × 10^17^ s at an ambient temperature of 298 K. This finding underscores the exceptional O_3_ adsorption potential of SnSe even at room temperature. The O_3_ molecules’ desorption time, however, drastically decreases to 244.91 s as the temperature steadily rises to 598 K. At a temperature of 698 K, the recovery time is observed to be 1.53 s. This observation implies that at higher temperatures, the adsorption of O_3_ on the SnSe monolayer becomes more reversible, enabling a rapid recovery to the initial state and demonstrating excellent dynamic response performance.

### 3.4. Sensitivity

To gain insight into the sensing capabilities of SnSe towards ozone, the sensitivity (S) of SnSe is calculated using the following equation:

(5) $$S=\left|I-I_{0}\right|/I_{0}$$

where I and I_0_ are the currents across the scattering region when adsorbed with O_3_ and in their pristine condition, respectively.

**Figure 6:**
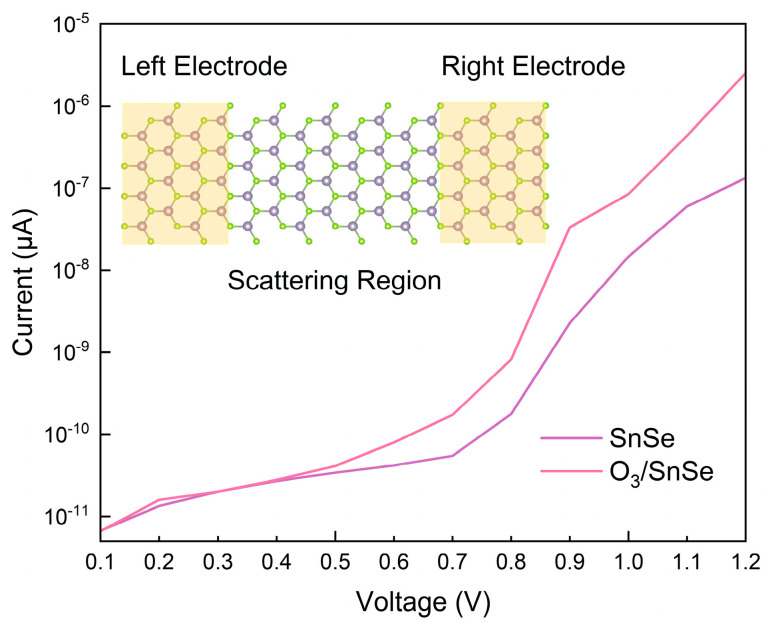
The current-voltage characteristics of pristine and O_3_-adsorped SnSe monolayers.

**Figure 7:**
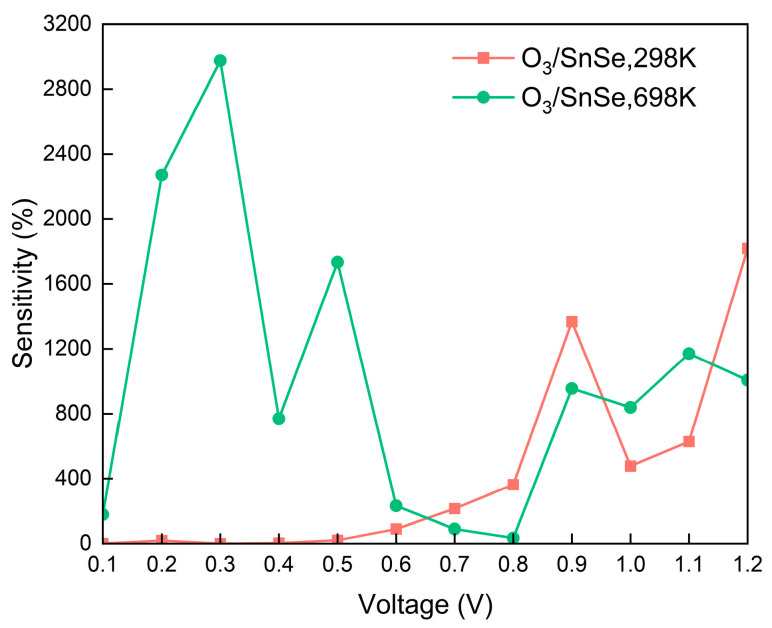
The sensitivity of O_3_-adsorped SnSe monolayers at temperatures of 298 K and 698 K.

An examination of the current-voltage (I–V) characteristics appears in Figure 6, which clarifies that O_3_ adsorption has very little effect on the semiconductor surface current at lower bias voltage ranges (≤0.6 V). This finding points to a slight difference in current values between the O_3_ adsorption and non-adsorption states. This suggests that at low voltage driving, O_3_ adsorption does not significantly alter the carrier transport characteristics of the semiconductor interface. Substituting the calculated current values into Equation (5) yields the sensitivity-voltage curve. As depicted in Figure 7, the sensitivity-voltage curve of SnSe for O_3_ at room temperature indicates that within the 0–0.6 V range, the sensitivity of SnSe to O_3_ detection remains relatively low and is consistently below 100%.

However, within the bias voltage range of 0.7 to 1.2 V, the influence of adsorbed O_3_ molecules on the surface current becomes more pronounced, resulting in SnSe exhibiting a sensitivity to O_3_ exceeding 200%. At a bias voltage of 1.2 V, the current response of the O_3_-adsorbed surface exhibits a marked enhancement, reaching a value of 2.54 × 10^−6^ μA. This represents an approximately one-order-of-magnitude increase compared to the current measured for the unadsorbed surface (1.33 × 10^−7^ μA), indicating a high degree of sensitivity. Consequently, the sensor system exhibits a remarkable sensitivity of 1817.57% at a working voltage of 1.2 V. This strongly proves that under high voltage excitation, the interaction between O_3_ and the semiconductor interface will greatly promote charge transfer and induce significant resistance changes, thus enabling the SnSe monolayer to have a highly sensitive detection capability for O_3_ molecules.

To comprehensively assess the detection capability of SnSe for O_3_ across different temperatures, the impact of temperature variations on the current-voltage (I–V) characteristics and sensitivity was investigated. The I–V curves at 298 K and 698 K are presented in Figure S1. As illustrated in Figure S1, increasing the temperature from 298 K to 698 K results in a significant rise in the current of SnSe post-O_3_ adsorption within the low bias voltage range (0–0.6 V), markedly surpassing the current of SnSe without O_3_ adsorption. Additionally, at 0.9–1.2 V, the current of SnSe post-O_3_ adsorption is distinctly higher than that of SnSe without O_3_ adsorption. Figure 7 illustrates that SnSe exhibits a sensitivity to O_3_ exceeding 200% at bias voltages of 0.2–0.6 V and 0.9–1.2 V. This observation suggests that, at elevated temperatures, the operational voltage range for O_3_ detection by SnSe extends to the lower voltage range of 0.2–0.6 V.

In addition to considering the influence of operating temperature, the selectivity of sensors towards the target gas in the presence of interfering gases is crucial for practical applications. To assess the selectivity of SnSe for O_3_ detection, the I–V curves and sensitivities of SnSe upon exposure to O_3_ and common air gases (CO_2_ and O_2_) are compared in Figures S2 and S3. As shown in Figure S2, the current of CO_2_-adsorbed SnSe within the bias voltage range of 0.1–1.2 V is significantly lower than that of O_3_-adsorbed SnSe, indicating minimal interference from CO_2_ in O_3_ sensing. However, in the bias range of 0.1–0.7 V, the current of O_2_-adsorbed SnSe exceeds that of O_3_-adsorbed SnSe, leading to interference in O_3_ detection. Therefore, 0.1–0.7 V is not an optimal bias range for SnSe-based O_3_ sensing. At bias voltages greater than or equal to 0.8 V, the current of O_3_-adsorbed SnSe surpasses that of O_2_-adsorbed SnSe. The sensitivity curves in Figure S3 further demonstrate that within the bias range of 0.8–1.1 V, the sensitivity of O_2_-adsorbed SnSe remains below 90%, while the sensitivity of O_3_-adsorbed SnSe consistently exceeds 300%. At a bias voltage of 1.2 V, the sensitivity of O_2_-adsorbed SnSe increases to 262.15%, while the sensitivity for O_3_ detection is approximately 6.9 times higher, reaching 1817.57%, and far exceeding that of O_2_-adsorption. These results confirm that SnSe exhibits high selectivity for O_3_ detection in the presence of common air gases (O_2_ and CO_2_) within the bias range of 0.8–1.2 V.

From the analysis presented, it is evident that within the bias voltage range of 0.9–1.2 V, SnSe can detect O_3_ across various temperatures without interference from high concentrations of ambient gases. Consequently, SnSe shows promise as a highly selective, highly sensitive, and reusable material for O_3_ sensing.

### 3.5. Comparison

In this study, a comparative analysis of the SnSe monolayer with previously reported O_3_ nanosensors is conducted, as detailed in Supplementary Table S1. In terms of electron transfer, the charge transfer amounts when O_3_ interacts with stanene, B-doped stanene [12], BN fullerene-like nanocages [4], and Ni-decorated B_12_N_12_ nanocages [3] are 0.557 e, 0.548 e, 0.5 e, and 0.789 e, respectively. These values are all significantly lower than the 0.881 e charge transfer value observed at the O_3_-SnSe interface. Compared to other two-dimensional materials such as MoS_2_ [29] and SnS [14], SnSe exhibits an adsorption energy for O_3_ that is 5.1 times and 1.5 times greater, respectively. Additionally, the electron transfer amount for SnSe is 7.0 times and 1.1 times higher than that of MoS_2_ and SnS [14]. Our findings reveal that the interaction between SnSe and O_3_ is significantly stronger compared to other two-dimensional materials, while maintaining excellent reusability.

Notably, a greater chemical connection between the gas molecule and the surface is usually indicated by a larger charge transfer between SnSe and O_3_, which results in increased sensor sensitivity. Graphene oxide [11] has the highest documented sensitivity to O_3_ for two-dimensional materials at 860%. This work, however, reveals that SnSe has a sensitivity of 1817.57% to O_3_, which is almost 2.11 times higher than that of graphene oxide. This suggests that SnSe has the potential to be a highly sensitive O_3_ detector.

## 4. Conclusions

Our findings reveal that the SnSe monolayer exhibits remarkable potential as a high-performance O_3_ sensing material. The calculated adsorption energy of −1.826 eV indicates a strong and spontaneous adsorption of O_3_ molecules on the SnSe surface, suggesting efficient capture at room temperature. O_3_ adsorption significantly alters the electronic structure of the SnSe monolayer, inducing a substantial band gap narrowing from 2.228 eV to 0.790 eV. This phenomenon translates to a significant enhancement in electrical conductivity, which is a crucial factor for sensor response. Bader charge analysis confirms a substantial charge transfer of 0.881 electrons from the SnSe monolayer to the O_3_ molecule, signifying a strong interaction and high adsorption stability. The recovery time for O_3_ desorption from the SnSe monolayer is 244.91 s, demonstrating excellent dynamic response performance. Notably, the SnSe monolayer demonstrates an exceptional sensitivity of 1817.57% towards O_3_ gas at a working voltage of 1.2 V, surpassing the previously reported two-dimensional materials (graphene oxide, 860%). This superior sensitivity can be attributed to the strong charge transfer between SnSe and O_3_ molecules. In conclusion, the SnSe monolayer emerges as a promising candidate for the development of highly sensitive and environmentally friendly O_3_ sensors.

## Data Availability

The original contributions presented in the study are included in the article/Supplementary Materials, further inquiries can be directed to the corresponding authors.

## Supplementary Materials

The following supporting information can be downloaded at: https://www.mdpi.com/article/10.3390/mi15080960/s1, Figure S1. The sensitivity for intrinsic and O_3_-adsorbed SnSe monolayers at temperatures from 298 to 698 K. Figure S2. Current-voltage characteristics of SnSe monolayers without adsorbed gas molecules and with adsorbed O_2_, O_3_, and CO_2_. Figure S3. Voltage-dependent sensitivity curves of SnSe monolayers with adsorbed O_2_, O_3_, and CO_2_ molecules. Table S1. Comparison between this work with previous reports. References [1,3,4,11,12,14,29] are cited in the supplementary materials.
