# Supplementary material for: Density Functional Theory Provides Insights into β-SnSe Monolayers as a Highly Sensitive and Recoverable Ozone Sensing Material
Source: Micromachines (Basel). 2024 Jul 27;15(8):960. doi: 10.3390/mi15080960 (PMC11356510; doi:10.3390/mi15080960)
Supplement: Supplementary file 1 [file micromachines-15-00960-s001.zip › micromachines-3099518-supplementary.pdf]

Density Functional Theory Provides Insights into β-SnSe Monolayers as a Highly Sensitive and Recoverable Ozone Sensing Material

Jiayin Wu [1,2], Zongbao Li [3,4,*], Tongle Liang [5], Qiuyan Mo [6], Jingting Wei [1], Bin Li [1] and Xiaobo Xing [2,*]

[1] Department of Engineering Technology, Guangdong Open University, Guangzhou 510091, China; wujiayin@m.scnu.edu.cn (J.W.); jtwei@gdrtvu.edu.cn (J.W.); bli@gdrtvu.edu.cn (B.L.)
[2] Centre for Optical and Electromagnetic Research, South China Academy of Advanced Optoelectronics, South China Normal University, Guangzhou 510006, China
[3] Ministry of Education Key Laboratory of Textile Fiber Products, School of Materials Science and Engineering, Wuhan Textile University, Wuhan 430220, China
[4] School of Materials and Chemistry Engineering, Tongren University, Tongren 554300, China
[5] School of Artificial Intelligence, Guangdong Vocational College of Post and Telecom, Guangzhou 510630, China; liangtongle@gupt.edu.cn
[6] Big Data Engineering College, Kaili University, Kaili 556011, China; 2014020983@kluniv.edu.cn
* Correspondence: zongbaoli1982@163.com (Z.L.); xingxiaobo@scnu.edu.cn (X.X.)

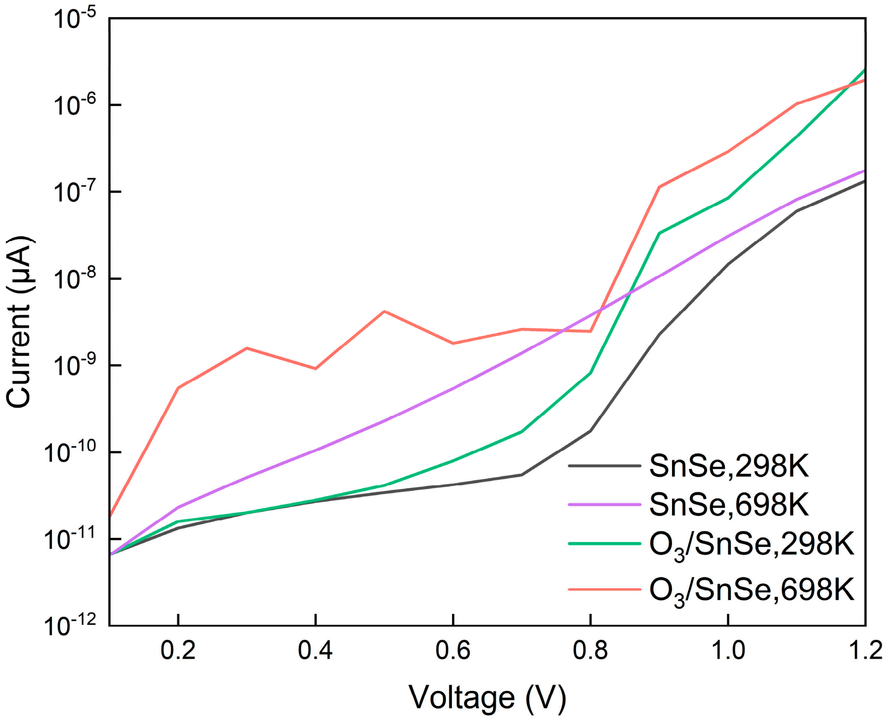

Figure S1. The sensitivity for intrinsic and O3-adsorbed SnSe monolayers at temperatures from 298 to 698 K

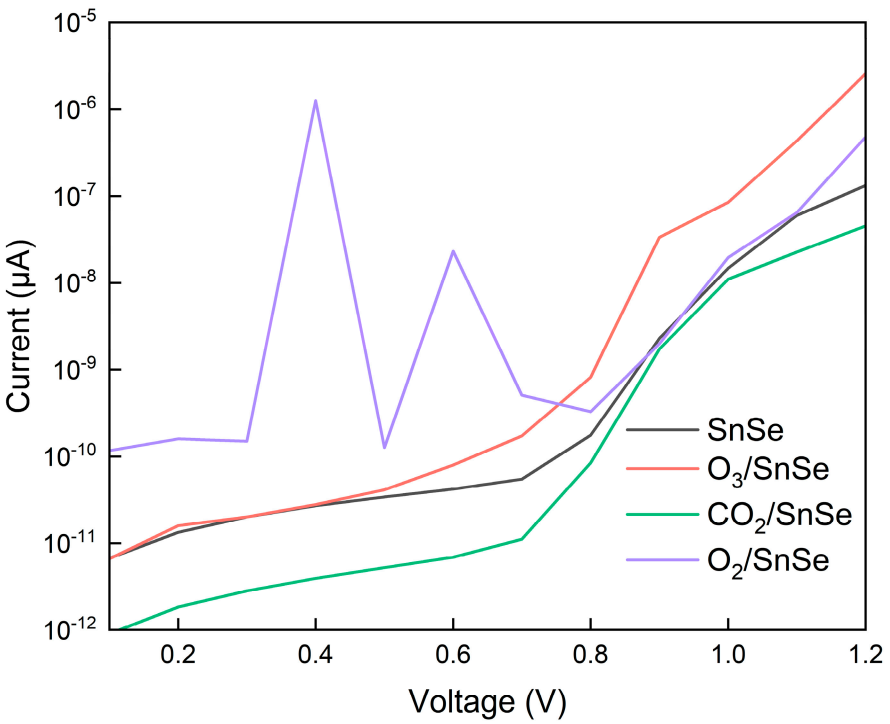

Figure S2. Current-voltage characteristics of SnSe monolayers without adsorbed gas molecules and with adsorbed O_2, O_3, and CO_2

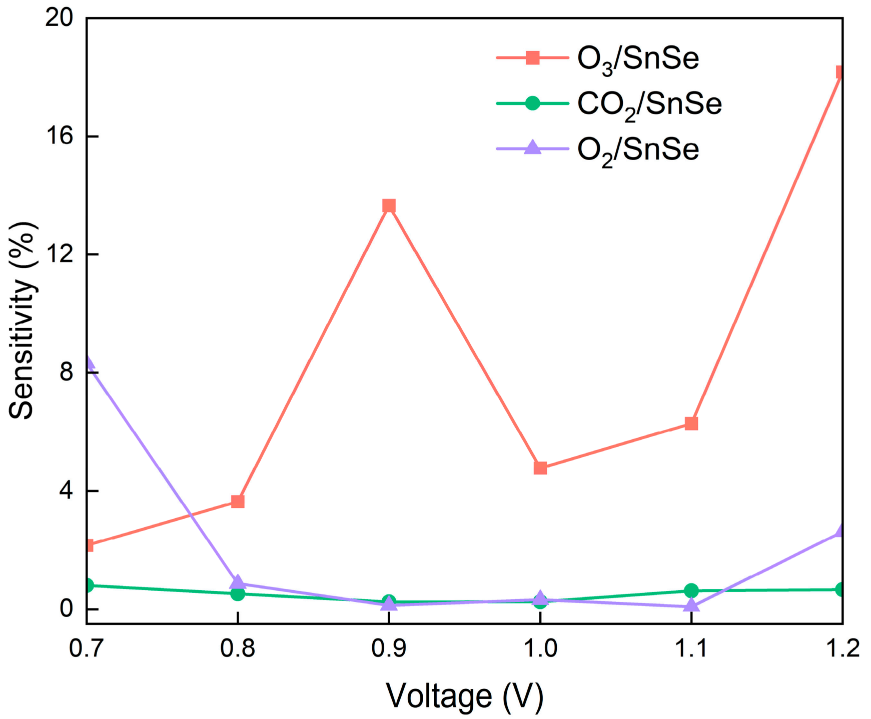

Figure S3. Voltage-dependent sensitivity curves of SnSe monolayers with adsorbed O_2, O_3, and CO_2 molecules

TABLE SI

Comparison between this work with previous reports

Sensing material	Adsorption energy E_{ad}(eV)	Charge transfer(e)	Recovery time, temperature	Sensitivity	Ref
SnSe	-1.826	0.881	1.53s, 698K	1817.57%	This work
MoS_2	-0.36	0.126	1.23×10^{-7} s, 298K[1]		[1]
SnS	-1.25	0.78	0.45s, 498K[1]		[2]
stanene	-2.06	0.557	74.79s, 698K[1]		[3]
B-doped stanene	-3.15	0.548	5.55×10^{9} s, 698K[1]		[3]
BN fullerene-like nano-cage	-1.8	-0.5	0.99s, 698K[1]		[4]
B--decorated $B_{12}N_{12}$ nanocages	-4.10 [2]	0.789			[5]
AlN nanotube	-1.88		3.75s, 698K[1]		[6]
graphene oxide				860%	[7]

[1] The recovery time was determined by extracting the adsorption energy data from the corresponding references and substituting it into Equation 4.

[2] The adsorption energy data from the references have been converted from kJ/mol to eV for consistency.

1. Abbasi, A.; Sardroodi, J.J. Adsorption of O_3, SO_2 and SO_3 Gas Molecules on MoS_2 Monolayers: A Computational Investigation. *Applied Surface Science* **2019**, *469*, 781–791, doi:10.1016/j.apsusc.2018.11.039.
2. Shukla, A.; Gaur, N.K. Adsorption of O_3, SO_3 and CH_2O on Two Dimensional SnS Monolayer: A First Principles Study. *Physica B: Condensed Matter* **2019**, *572*, 12–17, doi:10.1016/j.physb.2019.07.037.
3. Abbasi, A.; Sardroodi, J.J. The Adsorption of Sulfur Trioxide and Ozone Molecules on Stanene Nanosheets Investigated by DFT: Applications to Gas Sensor Devices. *Physica E: Low-dimensional Systems and Nanostructures* **2019**, *108*, 382–390, doi:10.1016/j.physe.2018.05.004.
4. Panahyab, A.; Soleymanabadi, H. Ozone Adsorption on a BN Fullerene-like Nano-Cage: A DFT Study. *MGC* **2016**, *15*, 347–354, doi:10.3233/MGC-160214.
5. Rad, A.S.; Ayub, K. O_3 and SO_2 Sensing Concept on Extended Surface of $B_{12}N_{12}$ Nanocages Modified by Nickel Decoration: A Comprehensive DFT Study. *Solid State Sciences* **2017**, *69*, 22–30, doi:10.1016/j.solidstatesciences.2017.05.007.
6. Kamalinahad, S.; Solimannejad, M.; Shakerzadeh, E. Sensing of Ozone (O_3) Molecule via Pristine Singe-Walled Aluminum Nitride Nanotube: A DFT Study. *Superlattices and Microstructures* **2016**, *89*, 390–397, doi:10.1016/j.spmi.2015.11.032.
7. Singh, S.; Goswamy, J.K.; Sapra, G.; Sharma, P. Sensitivity and Selectivity Analysis of Toxic Gases NO_2, SO_2, O_3, Cl_2, $(CH_3)_2NH$, CH_3NH_2, NH_3, HCl, CH_2CHCl and ClO_2 on GO Sheet Platform for Environmental Sustainability: A DFT Prediction. *Sensors and Actuators A: Physical* **2022**, *347*, 113899, doi:10.1016/j.sna.2022.113899.
